# BMI status relative to international and national growth references among Pakistani school-age girls

**DOI:** 10.1186/s12887-021-03017-z

**Published:** 2021-12-01

**Authors:** Rizwan Qaisar, Asima Karim

**Affiliations:** 1 Department of Basic Medical Sciences, College of Medicine, University of Sharjah, Sharjah, UAE; 2 Department of Physiology & Cell Biology, University of Health Sciences, Lahore, Pakistan

**Keywords:** Underweight, Overweight, Obesity, Schoolgirls, Punjab

## Abstract

**Background:**

A sizable proportion of school-going children from developing countries has abnormal growth parameters, often not standardized with international reference values. We aimed to assess the prevalence of underweight, overweight, and obesity in the schoolgirls of Punjab according to international and local references.

**Methods:**

In this population-based cross-sectional study, 10,050 school-going girls aged 8–16 years from 12 districts of northern, central, and southern Punjab were recruited. Estimates of normal weight, underweight, overweight and obesity were calculated in the girls according to three international BMI references including centers for disease control (CDC) 2000, the international obesity task force (IOTF) 2012 and world health organisation (WHO) 2007 in addition to a local reference for the population under study. We used Cohen’s kappa statistics to analyse the agreement of our data with reference values.

**Results:**

There was marked overestimation of underweight (23.9%, 14.5%, 15.2% and 4.37%), slight underestimation of overweight (5.3%, 7.3%, 7.9% and 8.97%) and moderate underestimation of obesity (1.9%, 1.5%, 2.2% and 5.67%) according to CDC, IOTF, WHO and local reference, respectively. When the weight status of the study cohort was compared with the local data, we found comparable results in all four weight categories.

**Conclusion:**

We recommend population-wide further studies to estimate the prevalence of weight status in school-age girls for devising appropriate references and for planning strategies for public health policy and management.

## Background

There is an increasing prevalence of abnormal growth including weight gain and underweight in Southeast Asia [1]. Pakistan faces a dual burden of overweight/obesity and underweight due to abnormalities in nutritional status in childhood and adolescence. Overweight and obesity may be attributed to lifestyle transitions, whereby a switch to a sedentary lifestyle leads to a weight gain in populations that previously had a trend of underweight or normal weight [2, 3]. Childhood obesity is associated with nutrition-related chronic diseases such as diabetes, cardiovascular diseases, and cancer in adulthood [4, 5]. On the other hand, childhood malnutrition/underweight is also a predisposing factor for ill health in adults. Both the abnormalities in weight status during childhood and adolescence contribute to developing several cardiac and metabolic diseases in adulthood [4, 6, 7]. Therefore, estimates and intervention of underweight and overweight/obese in the school-aged population is an important prerequisite applying for preventive medicine in paediatrics and the general health of the population [8].

We have included only girls in our studies and present work is a part of a series of investigations where we have assessed other parameters also of the school-age Punjabi girls. In our society girls are a victim of gender inequality and other abuses of human rights thereby, emphasizing the need to characterise their health status. Adolescence in girls is considered high-risk to weight gain due to changes in body composition, insulin sensitivity, eating habits, and physical activity [9, 10]. In teenage girls, the chances of being underweight are also high due to a change in eating habits or improper diet intake because of being conscious about self-image. Weight gains and undernutrition are two common problems in school-going girls in Pakistan, with significant repercussions in later life. Unfortunately among school children, girls are more prone to obesity than boys owing to the inherent difference in the pattern of female hormones [11]. Weight gain during adolescence in girls places them at an increased risk of accumulating high body fat stores during the childbearing age. An effective way to combat obesity during adolescence is by adopting a healthy lifestyle and eating habits that have far-reaching outcomes on the health of female conception periods [9, 12, 13]. On the other hand, poor nutrition in teenage girls has harmful effects on the health outcomes of future generations [14] because being underweight for girls has a strong association with infertility [15]. Malnutrition in school-aged girls also negatively impacts their cognitive abilities and educational performance [16]. Malnutrition is widespread in developing countries in preschool girls and the condition worsens in the school years. Underweight is mainly related to undernutrition and several other factors which cause an imbalance in calorie intake and expenditure [17]. Due to the ill impacts of this dual burden of abnormal nutrition on population health, it is imperative to understand its prevalence in school-aged girls.

To devise proper interventions, the first important task is to identify the high-risk paediatric population facing two extremes of nutrition, and the ideal tool to estimate the weight extremes is by using BMI [18]. For the children aged 0 to 5 years the growth status is assessed by the universally accepted world health organisation (WHO) references [19]. However, for school-aged children and adolescents, there is no globally consensual single reference. Multiple studies on assessment of weight status in older children have no consensus on utilising a single reference. One out of three accepted references i.e. centers for disease control (CDC), the international obesity task force (IOTF), and WHO is typically recommended in research or clinical practice. The criteria for estimating weight status are almost similar by these references and differ in categorizing the percentages of children falling in each category. Therefore, the prevalence of several categories would vary by applying different references. Few studies conducted to estimate the growth status of Pakistani school children have utilised either WHO and/or CDC references to measure the prevalence of two extremes i.e. under- and over- nutrition in older children and have found conflicting results [20–26]. This indicates dire need to come to a consensus for using one reference and to generate cut-off values for the indigenous population. In most of these national studies WHO reference has been used. However, its application in South Asian school children has not been considered appropriate due to their metabolic differences [27]. For example, an underestimation of the prevalence of overweight and obese and overestimation for underweight has been reported in Pakistani schoolchildren according to WHO references [22]. Regional differences partly explain this discrepancy among populations, the methodology of constructing the BMI-for-age reference graphs, and the criteria used to define BMI cut-offs [8, 28, 29]. Therefore, to correctly characterize weight status in Pakistani school children, the population-specific BMI cut-offs need to be generated and validated as the interpretation of a child’s growth status depends upon the growth reference used [30]. Moreover, the local BMI cut-offs, when compared with these references will help identify the discrepancy or consistency in classifying nutritional abnormalities in schoolgirls of Punjab, Pakistan.

Differences in the prevalence of underweight, overweight, and obesity have been observed among different districts of Punjab [31]. These differences can partly explain differences in geography, nutritional status of schoolgirls, or differences in the risk factors either at the individual or area level [32]. It was found that the region influences childhood weight abnormalities more than individual characteristics [32]. Local data describing the regional differences as contributing factors for obtaining variable results in the prevalence of weight status is scarce. Regional differences are essential to consider while tackling overweight/obesity and underweight for considering resource allocation and to conclude the success of area-based policies to deal with these weight abnormalities. Punjab being the most populous province, more thorough profiling of school children is required in a region-specific manner before appropriate interventions can be implied. We recently compared the percentile values of selected schoolgirl population to international references [33]; however, the relative contribution of abnormalities of weight status to local percentile values is not known.

Additionally, these changes are not performed in a region-specific manner. In this study, we aimed to fill these gaps by exploring the comparability of our study population BMI references with CDC, WHO and IOTF in assessing the weight status of 8–16 years old schoolgirls of Punjab overall and to northern Punjab (NP), central Punjab (CP) and southern Punjab (SP). We hypothesised that our study populations have weight status which only partially correlates with other international references and vary between the different regions of the province of Punjab.

## Methods

### Study group

We conducted this cross-sectional study at the University of Health Sciences (UHS), Lahore in January 2015.

We obtained data from schoolgirls aged 8–16 years (*n* = 10,050) from private and public schools of 12 districts of NP, CP, and SP using stratified multi-stage cluster sampling technique. We divided the Punjab province into central, northern, and southern regions according to local climate, topography, and population variations. The study cohort included data from NP (*n* = 1355; 13.5%), CP (*n* = 6580;65.47%), and SP (*n* = 2115;21%). We stratified each region into districts using the probability proportional to size technique, and the relative proportion of adolescent population in each district [34]. The information about demography was obtained from the Pakistan census bureau and about location and types of schools was obtained from the related District Education Officers of each district. The schools were further classified into urban, semi-urban, and rural status, based on their geographical locations. The students were randomly selected based on the age criteria. The inclusion criteria were the presence of normal and healthy appearance, while the girls with past medical or surgical history, presence of congenital disorders (e.g., Prader-Willi syndrome) and related conditions were excluded. The age and date of birth of each participant were obtained from the school record. For simplicity, we rounded off the age to the nearest full year; for example, an age of 10 years refers to participants aged between 9.5–10.49 years. The study cohort includes a homogenous representation of different socioeconomic status. The Anthropometric data was collected by filling in the questionnaires during our visit to the school. An Institutional Review Board of the UHS provided the ethical approval (UHS/ERB/22546/2014) for this study. The data was collected after obtaining informed consent from the parents of girls.

#### Anthropometric measurements

All anthropometric measurements (weight and height) were obtained by trained medical assistants according to the CDC’s recommendations (anthropometry procedures manual 2007 by the National Health and Nutrition Examination Survey). We used the same measuring tools for all data recordings for quality assurance. All measurements were obtained in the morning hours between 08:00 and 11:00 to avoid the influence of circadian rhythm.

***Weight*** in Kilograms was measured using a digital scale (City scale, Fzc, UAE) to the nearest approximation of 0.2 Kg. The weighing scale was calibrated before each recording. All participants wore light clothing and were bare feet during the recordings.

***Standing Height*** was measured using a portable stadiometer (SECA 217, SECA, USA), with a vertical stand and an adjustable headpiece. All measurements were obtained to a nearest approximation of 0.1 cm, and the stadiometer was calibrated with a set of predefined lengths before each measurement. All participants maintained an upright body posture during measurements.

#### Statistical analysis

All data entry and analysis were performed using SPSS Version 15.0 (SPSS Inc. Chicago IL, United States, 2009). BMI calculated by the formula BMI = weight (kgs) / height (m^2^). Data were presented as Mean ± SD for ‘n’ where n representing the number of study participants. The outliers, i.e.*,* values above and below 4SD were considered outliers and not included in the analysis. The outliers were identified from the z-score plot of the parameter under consideration and excluded from the dataset. The four levels of weight status [normal weight, underweight, overweight, or obese] were expressed according to the cut-offs obtained from three international BMI references and the cut-offs obtained from the study participants. The three international BMI references used in this study were CDC, IOTF, and WHO.

The CDC 2000 reference contains data from 32,653 children aged 2 to 20 years of age. This reference encompasses United States surveys and additional data from some national surveys conducted until the year 1994. According to this international reference, the BMI-for-age < 5th percentile is considered UW, ≥ 85th percentile is considered OW and ≥ 95th percentile as Ob.

The IOTF 2012 reference contains data from 1,92,727 children aged 2 to 18 years of age. This reference encompasses data from six countries namely Hong Kong, Great Britain, Singapore, Netherlands, Brazil, and the United States of America through the national health surveys conducted between 1963 to 1993. According to this international reference, BMI at the age of 18 with a value of 18.5, 25, and 30 kg/m^2^ is considered underweight, overweight, and obese. Therefore, in girls, BMI-for-age < 16.5th percentile, ≥ 89.3rd percentile, and ≥ 98.6th percentiles are considered underweight, overweight, and obese, respectively [29].

The WHO 2007 reference contains data obtained from 22,917 children aged 5–19 years of age. This reference encompasses data from the three surveys conducted on the US national population between 1963 and 1974. In addition to this, the WHO 2007 reference also contains data retrieved from the Multicenter Growth Reference Study from 1997 to 2003. According to this international reference BMI-for-age < 2SD below the mean, > 1SD above the mean and > 2SD above the mean is considered underweight, overweight, and Obese, respectively [35].

We also obtained BMI cut-offs from our study group data using BMI-for-age < 2SD below the mean, > 1SD above the mean, and > 2SD above the mean considering as underweight, overweight, and obese respectively. To have good comparable data across the references, we compared the data of our study group of girls 8–16 years with age and sex-matched values obtained from three international references and then applied cut-offs obtained from our data and estimated all the four weight categories in the study group. The incidences of normal weight, underweight, overweight, and obese were calculated for the pooled data to estimate the overall incidence in the province and then in all the three substrata i.e. NP, CP, and SP individually. Prevalence of normal weight, underweight, overweight, and obesity in different regions of Punjab vs. international references and in between the different regions was compared by using a two-sample t-test for percent values. Cohen kappa statistic was applied to estimate the overall agreement between the study population cut-offs with all the three international references while classifying normal weight, underweight, overweight, and obese. Kappa < 0.6 was defined as the poor agreement, 0.6 to < 0.8 as moderate agreement, and ≥ 0.9 as an excellent agreement [36]. The incidence of various weight status categories was compared by using a two-sample t-test for percent values and *p* ≤ 0.05 was considered statistically significant.

## Results

**Fig. 1:**
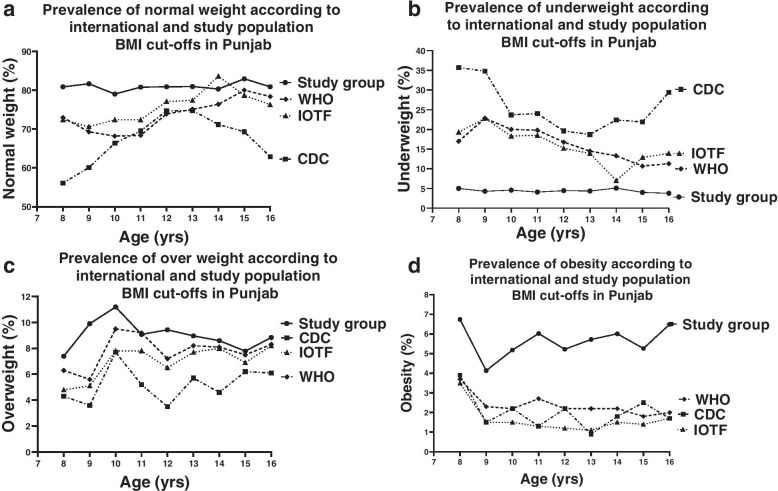
Age-related prevalence of **a** normal weight, **b** underweight **c** overweight and **d** obesity in girls 8–16 years using the CDC 2000 (interrupted line), IOTF 2012 (dotted line), WHO 2007 (dashed line) and study group (solid line) BMI references in Punjab (*n* = 10,050)

The mean age of 10,050 schoolgirls included in this study was of 12.7 ± 2.29 years (Mean ± SD). We investigated the weight status by categorizing the study population into three phases of development, including childhood (8–10 years), early- (11–13 years) and mid-adolescence (14–16 years) stages (Table 1), as described elsewhere. Next, we further dissected the weight status by sub-dividing each developmental category into age groups on yearly basis from 8 to 16 years. The overall prevalence of normal weight, underweight, overweight, and obesity was measured in the study population (*n* = 10,050) using three references and the study population cut-offs (Fig. 1). Overall, according to all the three references the percentages of normal weight girls of all age groups under study were comparable (68.9%, 76.8%, 74.6% according to CDC, IOTF, and WHO respectively). However, there was a notable prevalence of overweight (5.3%, 7.3%, 7.9% of our cohort were overweight according to CDC, IOTF and WHO respectively) and obesity (1.9%, 1.5%, 2.2% of our cohort were obese according to CDC, IOTF and WHO respectively) and a high prevalence of underweight (23.9%, 14.5%, 15.2% children were underweight according to CDC, IOTF and WHO respectively) especially in younger age groups (8, 9 years) in our paediatric population (Table 2). When we applied the local BMI cut-offs obtained from our study population then the prevalence of normal weight population was significantly higher (81%; *p*<0.05; Fig. 1a), the prevalence of overweight and obesity was higher (8.97% and 5.67% respectively; *p*<0.05; Fig. 1c & d) and a notably lower prevalence of underweight (4.37%; *p*<0.05; Fig. 1b) was found in comparison with the three international references. The estimates of the prevalence of growth status categories varied broadly between the three references. However, notably when the study group cut-offs were applied, the estimates for the prevalence of all weight categories displayed comparable results (Table 2). We compared BMI values from our cumulative study cohort with CDC, WHO, and IOTF references by applying kappa correlation. The study population had a poor agreement with WHO and IOTF, respectively (*κ* = 0.44; 0.44), while the correlation between the study population and CDC had the least agreement (*κ* = 0.27).

**Table 1:** Relative proportion of girls of four weight status categories (underweight, normal weight, overweight and obesity) during childhood (8–10 years), early- (11–13 years) and mid-adolescence (14–16 years) of age using age- and gender-specific BMI cut-offs from the Centres for Disease Control and Prevention (CDC), International Obesity Task Force (IOTF), World Health Organization (WHO) and the study group in the girls of Punjab; (*n* = 10,050)

***BMI Status Categories***	Childhood	Early adolescence	Mid-adolescence
**CDC reference**			
**Underweight (%)**	30.1	20.34	25.38
**Normal weight (%)**	61.93	73.31	66.98
**Overweight (%)**	5.58	4.84	5.69
**Obese (%)**	2.39	1.51	1.95
**IOTF reference**			
**Underweight (%)**	19.96	15.49	11.66
**Normal weight (%)**	71.90	76.03	79.02
**Overweight (%)**	6.22	7.32	7.79
**Obese (%)**	1.92	1.16	1.53
**WHO reference**			
**Underweight (%)**	20.16	16.6	11.71
**Normal weight (%)**	69.71	73	78.17
**Overweight (%)**	7.49	8.12	8.02
**Obese (%)**	2.59	2.32	2
**Study group reference**			
**Underweight (%)**	4.59	4.31	4.21
**Normal weight (%)**	80.31	80.90%	81.28
**Overweight (%)**	9.86	9.14	8.48
**Obese (%)**	5.24	5.65	6.03

**Table 2:** Incidence of four weight status categories (underweight, normal weight, overweight and obesity) using age- and gender-specific BMI cut-offs from the Centres for Disease Control and Prevention (CDC), International Obesity Task Force (IOTF), World Health Organization (WHO) and the study group in the girls of Punjab; (*n* = 10,050)

**CDC reference**										
***BMI Status Categories***	**8 y**	**9 y**	**10 y**	**11 y**	**12 y**	**13 y**	**14 y**	**15 y**	**16 y**	**Overall**
**Underweight (%)**	35.7	34.8	23.7	24	19.6	18.7	22.4	21.9	29.4	23.9
**Normal weight (%)**	56.1	60.1	66.4	69.5	74.7	74.8	71.2	69.3	62.9	68.9
**Overweight (%)**	4.3	3.6	7.7	5.2	3.5	5.7	4.6	6.2	6.1	5.3
**Obese (%)**	3.9	1.5	2.2	1.3	2.2	0.9	1.8	2.5	1.7	1.9
**IOTF reference**										
**Underweight (%)**	19.3	22.8	18.3	18.5	15.2	13.9	7	12.9	13.9	14.5
**Normal weight (%)**	72.4	70.6	72.4	72.4	77.1	77.4	83.6	78.7	76.3	76.8
**Overweight (%)**	4.8	5.1	7.8	7.8	6.5	7.7	8	6.9	8.2	7.3
**Obese (%)**	3.5	1.5	1.5	1.3	1.2	1.1	1.5	1.4	1.7	1.5
**WHO reference**										
**Underweight (%)**	17	22.8	20	19.8	16.8	14.5	13.3	10.7	11.3	15.2
**Normal weight (%)**	73	69.3	68.2	68.4	73.9	75.1	76.4	80	78.4	74.6
**Overweight (%)**	6.3	5.6	9.5	9.2	7.2	8.2	8.1	7.5	8.3	7.9
**Obese (%)**	3.7	2.3	2.2	2.7	2.2	2.2	2.2	1.8	2	2.2
**Study group reference**										
**Underweight (%)**	5	4.29	4.59	4.08	4.44	4.35	5.09	4	3.77	4.37
**Normal weight (%)**	80.87	81.68	79.03	80.82	80.9	80.97	80.32	82.95	80.91	81
**Overweight (%)**	7.39	9.9	11.19	9.08	9.43	8.96	8.59	7.79	8.83	8.97
**Obese (%)**	6.74	4.13	5.18	6.02	5.22	5.72	6.01	5.26	6.49	5.67

**Fig. 2:**
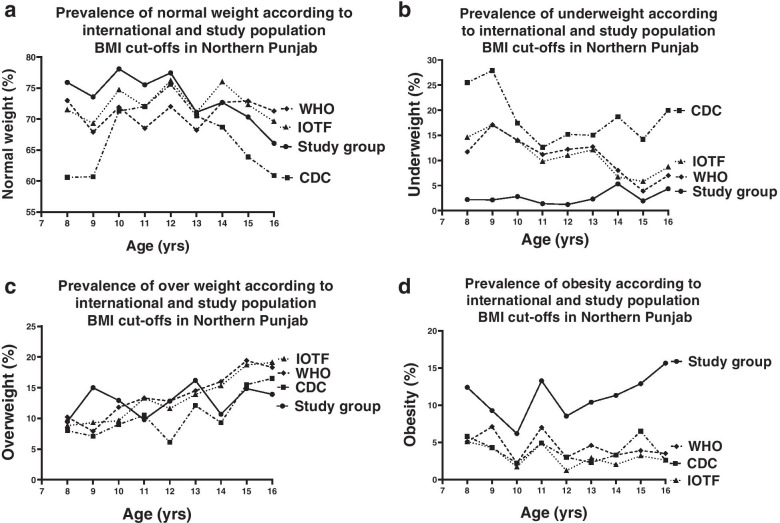
Age-related prevalence of **a** normal weight, **b** underweight **c** overweight and **d** obesity in girls 8–16 years using the CDC 2000 (interrupted line), IOTF 2012 (dotted line), WHO 2007 (dashed line) and study group (solid line) BMI references in the northern Punjab (*n* = 1355)

To dissect region-specific differences, we sub-categorized our study population into three regions of Punjab (NP, CP, and SP). We observed differences in the distribution of girls’ weight status categories across the study population subgroups when using three international references and local population cut-offs. Among the schoolgirls of NP (*n* = 1335), the estimates of percentages of normal weight children of all age groups accumulative were variable between all the references applied (63.7%, 67.4%, 70.9%, p<0.05; according to CDC, IOTF and WHO respectively) with the highest percentage found when local cut-offs were applied (73.65%; *p*<0.05; Table 3, Fig. 2a). Estimates of underweight prevalence varied greatly when values were compared after applying the three references between the several age groups analysed. Estimates for underweight prevalence were quite similar when using IOTF and WHO references (11.1% and 11% respectively), but estimates using CDC reference were noticeably higher (18.2%; *p*<0.05; Fig. 2b). The prevalence of underweight was strikingly lower in all the age groups when the study population cut-offs were applied (2.58%; *p*<0.05; Fig. 2b). Estimates of overweight were in agreement with the three international references when the local cut-offs were applied (10.3%, 13.1%, 13.7%, 12.92%; according to CDC, IOTF, WHO, and local cut-offs respectively, Fig. 2c). However, the incidence of obesity was significantly higher with the local cut-offs (10.85%, *p*<0.05) in comparison to all three references (3.8%, 3%, 4% according to CDC, IOTF and WHO respectively; Fig. 2d), with the highest prevalence among the16-year-old girls (15.65%).

**Table 3:** Incidence of four weight status categories (underweight, normal weight, overweight and obesity) using age- and gender-specific BMI cut-offs from CDC, IOTF, WHO and the study group in the girls of Northern Punjab; (*n* = 1355), Central Punjab; (*n* = *n* = 6580) and Southern Punjab; (*n* = 2115). ^*^p<0.05 vs CDC reference; ^#^*p*<0.05 vs IOTF reference; ^€^*p*<0.05 vs WHO reference; ^α^*p*<0.05 vs Northern Punjab reference; ^δ^*p*<0.05 vs Central Punjab reference. All analysis was conducted for all the age groups and weight status, using two-sample t-test between percent values

	Underweight (%)	Normal weight (%)	Overweight (%)	Obese (%)
**Northern Punjab**				
**CDC reference**	18.2	63.7	10.3	3.8
**IOTF reference**	11.1	67.4	13.1	3
**WHO reference**	11	70.9	13.7	4.4
**Study Population Reference**	2.58^*#€δ^	73.65^*#€δ^	12.92^*#€δ^	10.85^*#€δ^
**Central Punjab**				
**CDC reference**	26.9	67.4	4.3	1.5
**IOTF reference**	16.7	76.3	6	1.1
**WHO reference**	17.6	74.2	6.4	1.8
**Study Population Reference**	5.27^*#€α^	82.49^*#€α^	7.69^*#€α^	4.54^*#€α^
**Southern Punjab**				
**CDC reference**	18.3	74.4	5.3	2
**IOTF reference**	9.8	80.9	7.6	1.7
**WHO reference**	10.6	78.2	8.9	2.3
**Study Population Reference**	2.7^*#€δ^	81.04^**#€α^	10.4^*#€αδ^	5.86^*#€αδ^

**Fig. 3:**
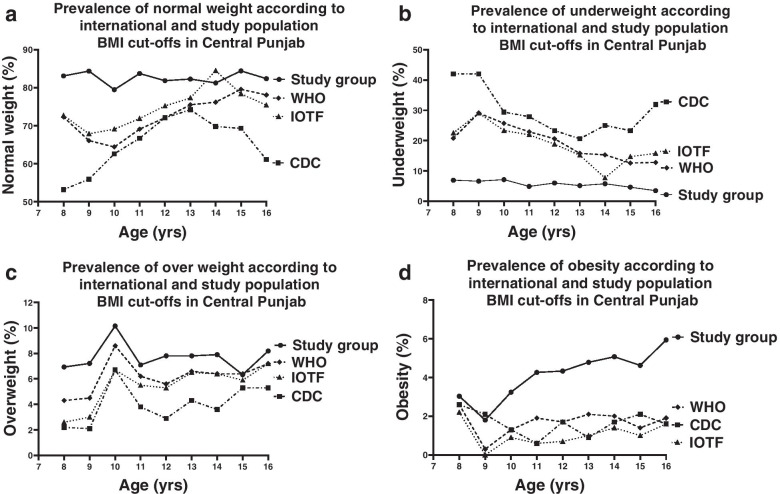
Age-related prevalence of **a** normal weight, **b** underweight **c** overweight and **d** obesity in girls 8–16 years using the CDC 2000 (interrupted line), IOTF 2012 (dotted line), WHO 2007 (dashed line) and study group (solid line) BMI references in central Punjab (*n* = 6580)

Estimates of normal weight varied markedly in the girls of CP (*n* = 6580) across all four references applied (67.4%, 76.3%, 74.2% according to CDC, IOTF and WHO respectively) and the highest incidence of normal weight girls was found with local cut-offs (82.49%, *p*<0.05, Table 3, Fig. 3a). Estimates of underweight prevalence were very high across the three references, with the highest estimates obtained with CDC reference (26.9%, 16.7%, 17.6%, according to CDC, IOTF and WHO respectively, Fig. 3b). Strikingly low estimates of underweight prevalence in all the age groups were observed with the application of local cut-offs (5.27%, *p*<0.05). Estimates for overweight and obesity prevalence were comparable (4.3%, 6%, 6.4% and 1.5%, 1.1%, 1.8% for overweight and obesity respectively, Fig. 3c &d) when using CDC, IOTF and WHO respectively, while prevalence estimates of overweight and obesity were found to be noticeably higher using local cut-offs (7.69%, 4.54% respectively).

**Fig. 4:**
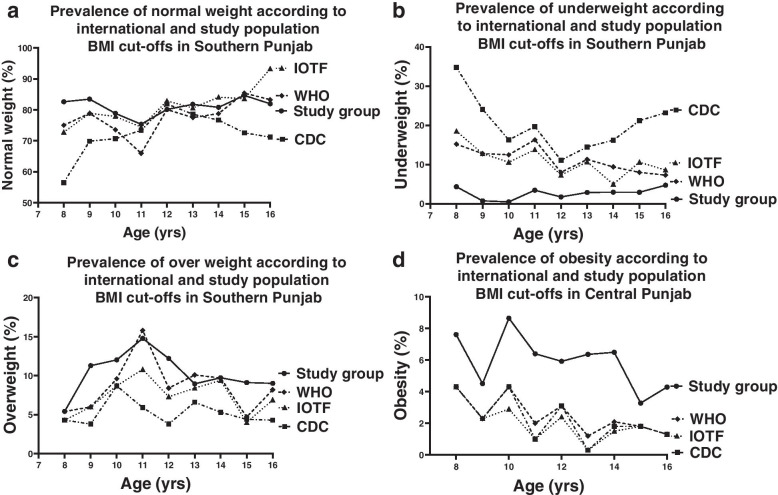
Age-related prevalence of **a** normal weight, **b** underweight **c** overweight and **d** obesity in girls 8–16 years using the CDC 2000 (interrupted line), IOTF 2012 (dotted line), WHO 2007 (dashed line) and study group (solid line) BMI references in southern Punjab (*n* = 2115)

Among the girls of SP (*n* = 2115), estimates of normal weight were comparable according to all the four references used (74.4%, 80.9%, 78.2%, 81.04% according to CDC, IOTF, WHO, and local references respectively, Table 3, Fig. 4a). We observed marked variation in the distribution of underweight estimates across the study groups. Underweight prevalence was found to be comparable when WHO and IOTF references were used (10.6%, 9.8% respectively, Fig. 4b), it was significantly higher with CDC reference (18.3%, *p*<0.05). The estimates of underweight were strikingly lower (2.7%, *p*<0.05) and consistency was observed among all the age groups of girls analysed when local cut-offs were applied (Fig. 4b). The overall prevalence of overweight (10.4%) and obesity (5.86%) was higher when local cut-offs applied as compared to CDC, IOTF, and WHO references (*p*<0.05; Fig. 4c & d).

**Fig. 5:**
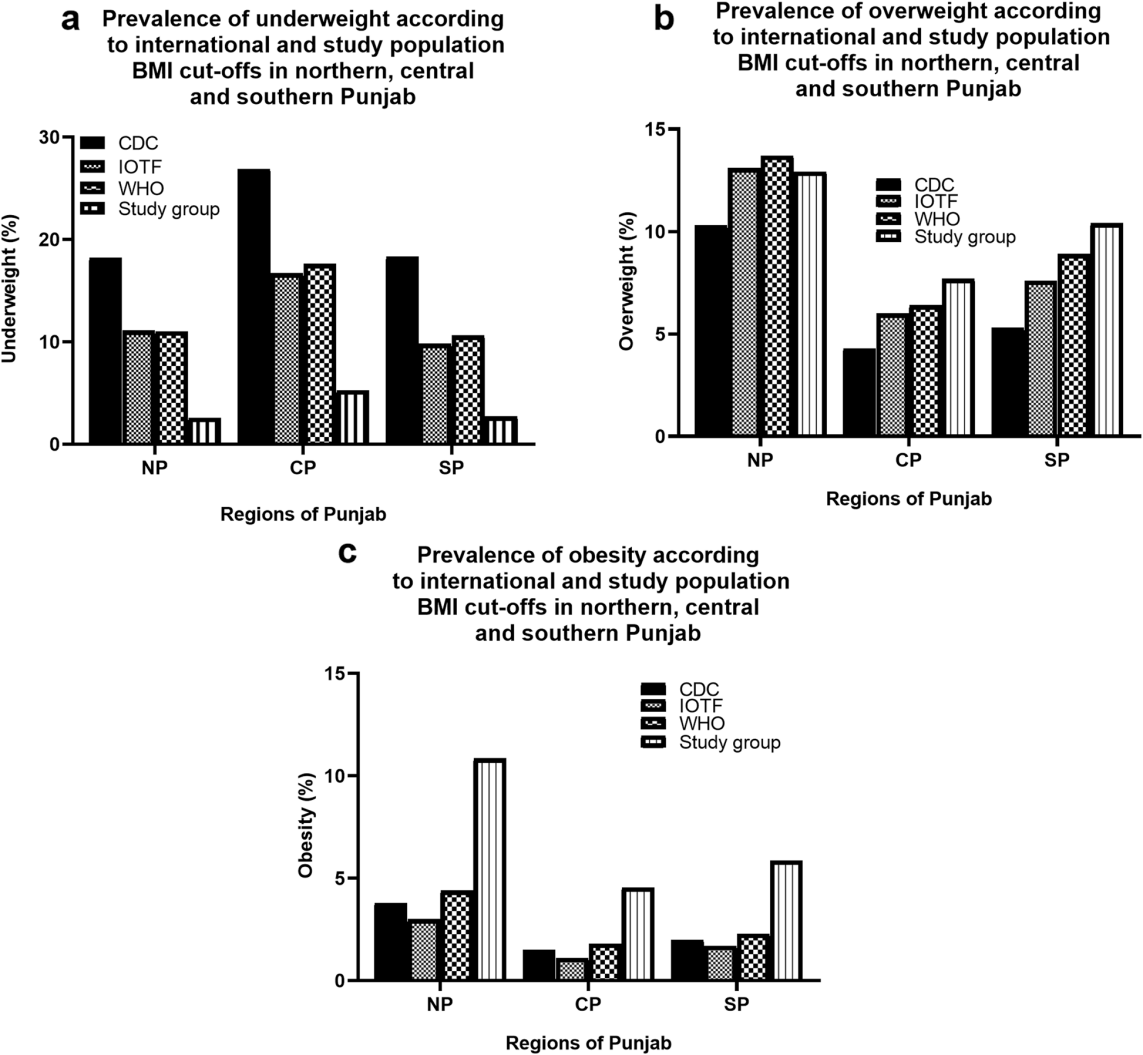
Prevalence of **a** underweight, **b** overweight, and **c** obesity in girls 8–16 years according to BMI cut-off values of international and national references in three regions of Punjab

When the three regions of Punjab i.e. NP, CP, and SP were compared for the prevalence of weight status by applying local BMI cut-offs, the schoolgirls of CP had the highest prevalence of being underweight compared to NP and SP (Fig. 5a). The girls belonging to NP had the highest prevalence of overweight and obese populations compared to central and southern Punjab (Fig. 5b & c).

Prevalence of normal weight, underweight, overweight and obesity according to CDC, IOTF, WHO, and local population cut-offs were compared by using the two-sample t-tests for percent and found significantly different among the girls of northern, central, and southern Punjab (*p* < 0.005). In summary, when CDC, IOTF, and WHO references have employed the prevalence of underweight was strikingly higher, estimates of overweight were moderately lower and obesity was remarkably lower than the local reference.

## Discussion

In this study of school-going girls aged 8–16 years from Punjab, we observed a very high prevalence of underweight and a significant burden of overweight and obesity among girls with the application of CDC, WHO, and IOTF references. The incidence of underweight was highest with CDC compared to WHO and IOTF and the incidence of overweight and obesity was generally comparable with all three references. Overall, the three references showed good agreement among themselves when applied to underweight and overweight status in our paediatric population (Fig. 1). Notably, we found a poor agreement when we compared the three international references (CDC, IOTF, and WHO) and the study population’s own BMI cut-offs. Our data suggest that the choice of BMI reference may significantly influence the estimates for the prevalence of underweight, overweight, and obesity, which can influence strategies adopted by the health resources to address these abnormalities. Additionally, these estimates may substantially affect the decisions by the paediatricians to recommend clinical advice and management.

Our results reinforce the existing problem of the dual burden of abnormal nutrition in the Pakistani population [25, 26, 37], where the prevalence of obesity is rising and underweight rising at the expense of the population with normal growth parameters. In recent years in Pakistan, there is little emphasis on estimating the prevalence of overweight in a region-specific manner, although the occurrences of overweight and obesity are increasing among school-going children [20, 23–26, 38, 39]. Few studies conducted to evaluate the weight problems used inconsistent references to define overweight and obesity undermining their usefulness in assessing and characterizing local populations. We addressed this problem by simultaneously applying all three primary international and one local reference values to regional populations of Punjab to allow for a streamlined clinical action and meaningful comparison of the incidence of abnormal weight status between the studies.

When WHO percentiles were applied to the Pakistani school-aged children there was an underestimation of overweight and obesity prevalence and overestimation for underweight [22]. Our study supports this trend and showed an underestimation of overweight/obesity and overestimation of underweight when CDC, IOTF, and WHO references were applied. Our results also indicate that the references applied to assess growth status are inconsistent even when utilized between demographic smaller groups. The trend of under- and overestimation could be explained in terms of differences in the sample size between the current study and the international references. The international references have been generated from many children and adolescent populations, and comparatively, our study has less sample size.

Our study results are in good agreement with the latest study conducted on 1244 Pakistani children aged 7–18 years [20], where the researchers applied the study population BMI cut-offs to estimate weight status in these children. We found most of the study population (81%) had the normal weight according to the study population cut-offs, which agrees with the previous study where they found 79.2% of children were normal weight. On the other hand, the prevalence of underweight in our study was 4.37% which is comparable with the incidence of 5.2% reported in the study mentioned above [20] but contrasts with findings from previous studies with an underweight prevalence of 29.7 and 27.3% according to the National Health Survey of Pakistan (NHSP) and Karachi surveys respectively [26]. In these national health surveys, the study population (5–14 years) was slightly younger than the present study (8–16 years) with a smaller sample size (*n* = 2074) than ours (*n* = 10,050). Further, they only applied WHO reference to estimate underweight incidence, while ignoring CDC and IOTF references. Moreover, we also noticed that when we applied WHO reference on our data the overall incidence of underweight was found to be 15.2%, which suggests that WHO reference overestimates underweight prevalence in our population.

The overall overweight prevalence of 8.97% was similar to that in the latest study on Pakistani children of 9.2% where the researchers applied indigenous BMI cut-offs [20]. This however differed from the NHSP and Karachi survey which reported incidences of overweight and obesity as 3 and 5.7% respectively [26], lower than the incidence in our study group. Similarly, the incidence of overweight in our paediatric population as reported in previous national studies is quite inconsistent and reported to be 17% (5–12 years) with WHO reference, 33% with IOTF reference [38]; 19.1% (11–15 years) with CDC reference [24]; 16% (10–14 years) with WHO reference [23]; 8% (10–14 years) with modified BMI cut-offs for Asian population [25]; 19% (6–17 years) with CDC reference. The overall prevalence of obesity in our 8–16 years old schoolgirls was 5.67%. This was comparable with the obesity incidence of 6.4% in the latest study conducted on Pakistani children 7–18 years of age where study population BMI cut-offs were applied [20]. This was in agreement with the incidence of obesity reported by the Karachi survey of 5.7% [26]. This incidence of obesity as reported by previous researchers in several studies conducted on Pakistani children showed inconsistent results [23, 24, 38] and differed from our study estimates. Additionally, we observed significant differences in the estimates of weight status when school-going girls from NP, CP, and SP were compared (Figs. 2, 3, 4 and 5 respectively). The incidence of overweight and obesity was found to be highest in NP, and the incidence of underweight was highest in the CP. We are not sure about the underlying factors contributing to these differences. However, these three regions of Punjab differ enormously in terms of climate, culture, geography, dietary patterns, and living styles and these factors can affect the growth of children and may lead to differences in estimates of abnormal nutrition incidences reported here.

This study highlights the importance of understanding the comparability of globally used references for anthropometric measurements when applied to our paediatric population and their degree of agreement with the study population’s cut-offs. Several countries have emphasized generating local anthropometric references based on their population data [22, 40, 41]. Data from such populations shows that it is not justifiable to utilise one reference as a universal one to all populations because the growth pattern of children differs significantly between populations. It is challenging to evaluate population-specific references in comparison with international references as there are no established criteria for comparison. The population-specific references must be robustly validated to assess their potential in predicting adverse health consequences in adulthood. Unfortunately, the available data on the health assessment of adults with local references is scarce and warrant the comparison with international references despite population-specific differences [42]. However, the importance of utilising international references for comparison cannot be eliminated because this can provide essential insights to assess the effect of factors such as ethnicity, culture, and region on the growth parameters of the human body. Although there is a consensus on the utilisation of international references in the younger age group, reference values are not well defined for the older children, especially the school-aged population.

Identifying the incidence of underweight and overweight/obese Punjabi schoolgirls will help devise appropriate health policies to reduce the ill effects of abnormal childhood growth in later life. Considering the importance of adequate health status of females on the next generations, this data will be valuable in a timely assessment of the challenge faced and to devise measurements accordingly. These estimates will provide paediatricians a valuable tool to establish appropriate treatments to combat the abnormal growth status in childhood. Timely evaluation of abnormal weight status will also assist in reducing the economic burden by avoiding undergoing expensive treatments of cardio-metabolic diseases because of childhood obesity or underweight. Moreover, identifying the weight status of teenage girls and devising appropriate strategies will help them boost their self-confidence and improve their self-image.

There are certain limitations to our study. First, the study was conducted on the girls and boys were not included in the study. Second, this study was conducted in Punjab, one of the four provinces of Pakistan, so other provinces also need to be considered in the future. Third, it was a cross-sectional study, thereby depicting the estimates of malnutrition i.e., underweight, overweight, and obesity, at a one-time point. Longitudinal studies are recommended to generate population-specific reference, trace the trend of abnormal weight status, and assess the future risk of cardiometabolic and other adverse outcomes in adulthood.

## Conclusions

In conclusion, we observed noticeable differences in estimating the incidence of abnormal nutrition for classification of underweight, normal weight, overweight, and obesity among the schoolgirls of Punjab aged 8–16 years of age when CDC, IOTF, WHO, and local references were applied to the study population. There was a notable incidence of overestimation for underweight and underestimation of overweight and obesity among the study population when the international references were applied compared to the study population’s references. The comparison with international references should be interpreted cautiously because they might potentially have a bias in their distribution towards lower values of body weight. Our results indicate the importance of conducting longitudinal studies to generate population-specific references. Estimation of weight status should be uniform throughout the population to initiate national policies to combat the dual burden of abnormal nutrition in our school-age girls.

## Data Availability

The datasets used and/or analysed during the current study are available from the corresponding author on reasonable request.

## Abbreviations

BMI: Body mass index
CDC: Centres for disease control and prevention
WHO: World health organization
IOTF: International obesity taskforce
IREC: Institutional ethical review committee
NHANES: National health and nutrition examination survey
